# Spontaneous rupture of the long head of the biceps tendon in a woman with hypothyroidism: a case report

**DOI:** 10.1186/s13256-015-0794-2

**Published:** 2016-01-13

**Authors:** K. Pantazis, N. D. Roupas, Andreas Panagopoulos, S. Theodoraki, A. Tsintoni, V. Kyriazopoulou

**Affiliations:** 1 Department of Shoulder and Elbow Surgery, University of Patras, 26500 Patras, Greece; 2 Division of Endocrinology, Diabetes and Metabolic Diseases, Department of Internal Medicine, University of Patras, 26500 Patras, Greece; 3 Department of Internal Medicine, University of Patras, 26500 Patras, Greece

**Keywords:** Hypothyroidism, Long head of biceps, Tendon rupture, Thyroid

## Abstract

**Background:**

Tendinitis can be a presenting complaint in hypothyroidism, with symptomatic relief being obtained by appropriate management of the primary thyroid deficiency. To the best of our knowledge no other cases of spontaneous rupture of the long head of the biceps tendon during uncontrolled hypothyroidism have yet been reported.

**Case presentation:**

This case report describes an unusual case of spontaneous rupture of the long head of the biceps tendon in a 48-year-old white woman with severe hypothyroidism. She described experiencing a sudden sharp pain and an audible pop in her right shoulder while using her personal computer. On physical examination she was positive for Yergason’s sign and a subsequent magnetic resonance imaging scan showed complete rupture of the long head of her biceps tendon. Laboratory tests revealed significantly elevated thyrotropin levels (>100 μIU/ml) and very low levels of both triiodothyronine (0.17 ng/ml) and free thyroxine (0.18 ng/dl). She was switched to a different thyroxin regimen with a progressive dosage increment. She declined surgical re-anchorage of the tendon but despite the discreet Popeye sign, her overall strength and shoulder function were satisfactory. After 2 months, she was found to be clinically euthyroid, having normal thyroid function tests (thyrotropin 2.95 μIU/mL, free thyroxine 1.07 ng/dl). At her last follow-up visit, 1 year post-injury, she reported nearly normal shoulder function in her daily activities and had a constant shoulder score of 93 points.

**Conclusions:**

The role of thyroid hormones in the synthesis and degeneration of collagen and in the proliferation and apoptosis of human tenocytes is discussed, providing a possible mechanism whereby hypothyroidism may lead to tendon tears. This report may have a greater impact among different subspecialties as it presupposes a high degree of awareness from internists, endocrinologists and orthopedic surgeons.

## Background

The thyroid hormones (THs) triiodothyronine (T_3_) and thyroxine (T_4_) play an essential role in the development and regulation of many tissues and organs and have profound metabolic effects during adult life. Although muscle involvement in a variety of forms (weakness, cramps, myalgia) is a common problem in both congenital and adult-onset hypothyroidism, very few data are available regarding the effect of thyroid dysfunction on connective tissue. Tendinitis can be the presenting complaint in hypothyroidism and symptomatic relief can be obtained by appropriate management of the primary thyroid deficiency [1]. Calcified tendinopathy has also been associated with thyroid dysfunction [2].

To the best of our knowledge there is no published data reporting the association of thyroid function with spontaneous tendon rupture. This case is therefore unusual and interesting because it describes a middle-aged woman with severe hypothyroidism diagnosed with shoulder rotator cuff impingement syndrome and spontaneous rupture of the long head of her biceps tendon.

## Case presentation

A 48-year-old white woman presented at the Emergency Department of our hospital complaining of the acute onset of severe pain in her right shoulder. She clearly reported no trauma or strenuous physical activity and denied any previous shoulder pain or functional impairment. She had been diagnosed with a multinodular goiter 10 years previously and had been treated with near-total thyroidectomy and subsequent replacement therapy with levothyroxine. Annual follow-up tests were performed by her endocrinologist and she stated that her thyroid function tests had been within the normal range during her most recent examination 3 months earlier. She was otherwise healthy and reported taking no other medication prior to the incident; in particular, she was not taking any steroids, statins, Ferrum (folic acid) or fluoroquinolone-based drugs.

**Fig. 1:**
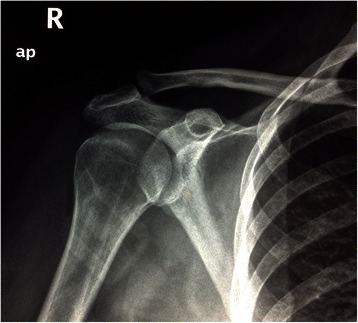
Anteroposterior X-ray of the patient’s right shoulder demonstrating mild proximal elevation of the head and moderate arthritis in her acromioclavicular joint

A physical examination of her right shoulder revealed a nearly normal range of motion and muscle strength but she had positive results from impingement tests. An anteroposterior X-ray of her shoulder was normal apart from a mild proximal elevation of the head and moderate arthritis in her acromioclavicular joint (Fig. 1). No evidence of calcified tendinitis was detected.

**Fig. 2:**
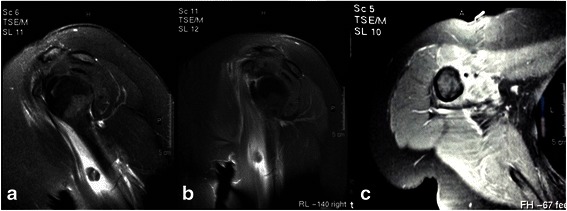
Magnetic resonance imaging scan of the patient’s shoulder. **a** T2/turbo spin echo /spectral presaturation with inversion recovery sagittal oblique view: fluid in her biceps tendon sheath with the long head of her biceps tendon torn and turned downwards. **b** T1 spectral presaturation with inversion recovery and gadolinium sagittal view: biceps tendon turned downwards. **c** Discontinuation of the long head of her biceps tendon in the sheath and edema at the region of subscapularis tendon

She was managed conservatively with cryotherapy, sling immobilization of her shoulder and non-steroidal anti-inflammatory drugs (NSAIDs). However, within a week she again attended our Emergency Department, presenting with extreme pain in her shoulder, fever up to 38.5 °C and mild symptoms of upper respiratory tract infection (nonproductive cough and nose dripping). She described a sudden sharp pain and an audible pop in her right shoulder while using her personal computer (PC). A physical examination revealed positive Neer, Hawkins and Yergason’s signs and a positive Jobe’s test. After orthopedic consultation she was admitted to our Internal Medicine Department for further evaluation. Laboratory analysis revealed significantly elevated thyrotropin (TSH) levels (>100 μIU/ml) and very low levels of both T_3_ (0.17 ng/ml) and free T_4_ (0.18 ng/dl). Of interest, her creatine phosphokinase (CPK) levels were found to be within the normal range. Two inflammation markers were also within the normal range: erythrocyte sedimentation rate (ESR) and C-reactive protein (CRP). Chest radiography, urine analysis and urine and blood cultures were negative. A magnetic resonance imaging (MRI) scan of her right shoulder revealed a complete rupture of the long head of her biceps tendon (Fig. 2a–c).

She was switched to a different thyroxin regimen with a progressive dosage increment. She was re-evaluated by our Out-patient Department 3 weeks after the hormone replacement treatment was started to discuss the possible surgical re-anchorage of her ruptured biceps tendon. She had significantly improved, having positive impingement tests only in the extremes of motion. Despite the discreet Popeye sign, the overall strength of her biceps tendon was satisfactory and she consequently decided against any surgical treatment. This prevented a proper histopathological examination during surgery and thus the confirmation of our clinical findings. After 2 months, she was re-evaluated by her endocrinologist and she was found clinically euthyroid, with tests showing normal thyroid function (TSH 2.95 μIU/mL, free T_4_ 1.07 ng/dl).

At her last follow-up visit, 1 year post-injury, she reported nearly normal shoulder function in her daily activities and had a constant shoulder score of 93 points.

## Discussion

Shoulder pain is a common presenting symptom observed at primary care offices and sports medicine clinics. Rotator cuff pathology is the most common finding among patients with shoulder pain asking for medical treatment [3]. In a recent systematic review (2004), Oh *et al*. [4] showed that rotator cuff problems accounted for more than 4.5 million clinician visits and approximately 40,000 surgeries every year in the USA. Rotator cuff impingement is primarily a disease of middle-aged and older patients. Tendinitis or tear of the long head of the biceps tendon has been associated with impingement pathology but the exact cause of tearing is unknown and probably multifactorial. Age, overuse, corticosteroid use, hyperthyroidism, subacromial impingement, heavy activities or acute injuries may all contribute to the development of long head tears. To the best of our knowledge, the present case report is the first to report hypothyroidism as a possible cause of subacromial impingement syndrome and complete tear of the long head of the biceps tendon.

Although there are no data to suggest an association between thyroid disorders and tendon tears, there is sufficient evidence to suggest a relationship between TH action and collagen metabolism [5, 6]. More specifically, hyperthyroidism is accompanied by increased rates of catabolism of both soluble and insoluble collagen, while hypothyroidism is characterized by decreased synthesis and degeneration of collagen. Hypothyroidism also inhibits epimerase, which results in reduced chondroitin sulfate and elevated hyaluronic acid, hence weakening the matrix. Moreover, it causes accumulation of glycosaminoglycans (GAGs) in the extracellular matrix (ECM), being involved in the pathogenesis of carpal tunnel syndrome during hypothyroidism and predisposing patients to tendon calcification [7].

THs have known effects at the cellular level on the proliferation and differentiation of bone and cartilage. The hypothyroid state appears to induce abnormalities in these tissues, which results in such clinical manifestations as epiphyseal dysgenesis, aseptic necrosis, possibly crystal-induced arthritis, and an arthropathy characterized by highly viscous non-inflammatory joint effusions primarily affecting the knees, wrists, and hands. Neuropathic and myopathic symptoms accompanying hypothyroidism may manifest as joint region abnormalities when in fact there is no underlying arthropathy [8, 9].

Apart from the relationship between TH action and collagen metabolism, a recent study demonstrated the presence of TH receptors in healthy and pathologic tendons, thus suggesting a possible role in the proliferation and apoptosis of human tenocytes [10]. Tenocytes are specialized fibroblasts which contribute to tendon ECM component homeostasis through a wide variety of complex mechanisms. *In vitro*, THs enhance tenocyte growth and counteract apoptosis in healthy tenocytes isolated from tendons in a dose-dependent and time-dependent manner [10]. These results reinforce the concept of a physiological role of THs in the homeostasis of tendons, providing a possible mechanism whereby hypothyroidism may lead to tendon tears.

The range of manifestations of myxedema includes stiffness, joint pain, joint swelling, calcium pyrophosphate dihydrate (CPPD) crystal deposition disease, popliteal cysts, ligamentous laxity and flexor tendon sheath thickening. Needle synovial biopsy studies have shown only mild inflammation in the thick synovium. These findings suggest that the musculoskeletal problems caused by hypothyroidism are treatable [11], despite the lack of unanimity regarding the course of musculoskeletal symptoms and signs after treatment of the thyroid dysfunction. Several authors have reported complete disappearance, while others have reported only a decrease in symptoms and signs. Kloppenburg *et al*. [12] concluded that musculoskeletal complaints due to thyroid dysfunction would remain present in half the patients even after restoration of thyroid dysfunction, and in the long run the vast majority of patients would have persistent or renewed musculoskeletal complaints. Clinical and electrophysiological studies suggest that hypothyroid patients do have a myopathy rather than a functional muscle disease [13].

Tendinitis has been reported as a frequent complaint in cases of hypothyroidism while symptomatic relief is obtained by the treatment of the primary deficiency. Symptoms can be protracted, resulting in lost working time and lower quality of life. The majority of patients respond well to either operative or non-operative treatment. Despite this, patients develop symptoms at a younger age, resist conservative treatment and more frequently undergo surgical treatment than patients with no associated endocrine disease [2].

## Conclusions

Spontaneous rupture of the long head of the biceps tendon is a rare complication of hypothyroidism. THs have known effects at the cellular level on the proliferation and differentiation of connective tissues and collagen formation. The hypothyroid state appears to induce abnormalities in these tissues, which results in clinical manifestations.

## Consent

Written informed consent was obtained from the patient for publication of this case report and any accompanying images. A copy of the written consent is available for review by the Editor-in-Chief of this journal.
